# Crystal structure of [Cu(tmpen)](BF_4_)_2_ {tmpen is *N*,*N*,*N*′,*N*′-tetra­kis­[(6-methyl­pyridin-2-yl)meth­yl]ethane-1,2-di­amine}

**DOI:** 10.1107/S2056989017004492

**Published:** 2017-03-31

**Authors:** Lin Chen, Yakun Guo, Gan Ren, Ge Sang

**Affiliations:** a Science and Technology on Surface Physics and Chemistry Laboratory, Jiangyou 621908, People’s Republic of China; b Institute of Materials, China Academy of Engineering Physics, Jiangyou 621908, People’s Republic of China

**Keywords:** crystal structure, copper, catalysis, CO_2_ reduction, electrochemistry

## Abstract

The mononuclear copper title complex {*N*,*N*,*N*′,*N*′-tetra­kis­[(6-methyl­pyridin-2-yl)meth­yl]ethane-1,2-di­amine-κ^6^ *N*}copper(II) bis­(tetra­fluorido­borate), [Cu(C_30_H_36_N_6_)](BF_4_)_2_, is conveniently prepared from the reaction of Cu(BF_4_)_2_·6H_2_O with *N*,*N*,*N*′,*N*′-tetra­kis­[(6-methyl­pyridin-2-yl)meth­yl]ethane-1,2-di­amine (tmpen) in aceto­nitrile at room temperature in air. The complex shows a distorted octa­hedral environment around the Cu^II^ cation (site symmetry 2) and adopts the centrosymmetric space group *C*2/*c*. The presence of the 6-methyl substituent hinders the approach of the pyridine group to the Cu^II^ core. The bond lengths about the Cu^II^ atom are significantly longer than those of analogues without the 6-methyl substituents.

## Abstract 2

The mononuclear copper complex {*N*,*N*,*N*′,*N*′-tetra­kis­[(6-methyl­pyridin-2-yl)meth­yl]ethane-1,2-di­amine-κ^6^ *N*}copper(II) bis­(tetra­fluorido­borate) shows a distorted octa­hedral environment around the Cu^II^ cation. The presence of the 6-methyl substituent hinders the approach of the pyridine group to the Cu^II^ core. The bond lengths about Cu^II^ are significantly longer than those of analogues without the 6-methyl substituents.

## Chemical context   

Copper complexes with polypyridine ligands are of great inter­est in catalytic reactions. For example, the copper-based complex CuBr[*N*,*N*,*N*′,*N*′-tetra­kis­(2-pyridyl­meth­yl)ethyl­ene­di­amine] (TPEN) is reported as a versatile and highly active catalyst for acrylic, methacrylic and styrenic monomers (Tang *et al.*, 2006 ▸). Copper(II) *N*-benzyl-*N*,*N*′,*N*′-tris­(pyridin-2-ylmeth­yl)ethyl­enedi­amine (bztpen) displays high catalytic activity for electrochemical proton reduction in acidic aqueous solutions, with a calculated hydrogen-generation rate constant (k_obs_) of over 10000 s^−1^ (Zhang *et al.*, 2014 ▸). [Cu_2_(m-xpt)_2_(NO_3_)_2_](PF_6_)_2_ [m-xpt = *m*-xylylenebis(pyridyl­triazole)] can selectively capture CO_2_ from air and reduce it to oxalate, in the form of an oxalate-bridged complex (Pokharel *et al.*, 2014 ▸). Generally, the reduction of a metal complex is accompanied by ligand dissociation (reductive dissociation), which is able to give the appearance of an open site for catalytic reaction. Herein, we describe the structure of the title complex, **1**.
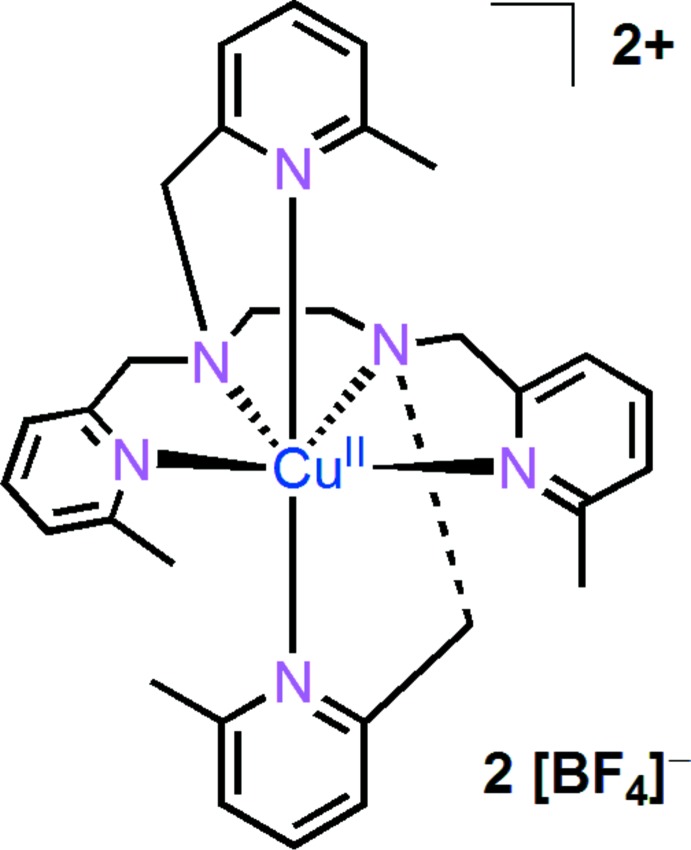


## Structural commentary   

**Figure 1:**
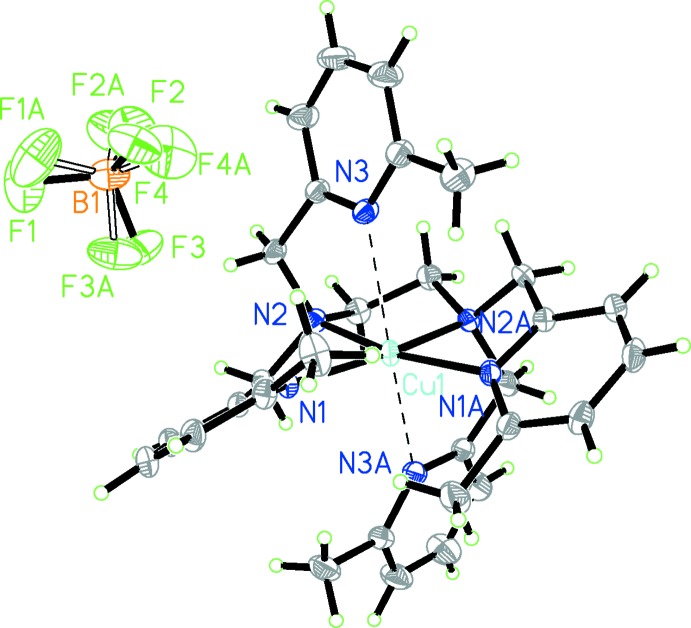
The molecular entities in the structure of complex **1**. Atoms N1*A*, N2*A* and N3*A* are generated by the symmetry operation −*x*, *y*, 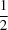 − *z*.

In the title complex (Fig. 1 ▸), the coordination sphere of the copper(II) atom is distorted octa­hedral, presumably as a result of the introduction of the 6-methyl substituent. Two pyridine nitro­gen atoms (N1, N1′) and two amino nitro­gen atoms (N2, N2′) form the equatorial planar coordination, while the apical positions are occupied by the other two pyridine nitro­gen atoms (N3, N3′). The Cu^II^ ion lies almost in the equatorial plane. The Cu—N bond lengths for the two axial pyridine-nitro­gen atoms [Cu—N3 = 2.5742 (13) Å] are significantly longer than those for the other four nitro­gen atoms [Cu—N1 = 2.0571 (13), Cu—N2 = 2.0311 (13) Å]. The long Cu—N3 distance indicates a weak connection between copper and pyridine, which is apt to dissociate under reductive conditions (Tang *et al.*, 2006 ▸). As a result of steric hindrance from the methyl group, the N3—Cu1—N3′ bond angle is not linear but rather 164.94 (5)°. The pyridine rings in the equatorial plane (N1/C2–C6 and N1′/C2′–C6′) subtend a dihedral angle of 35.03 (9)°.

**Figure 2:**
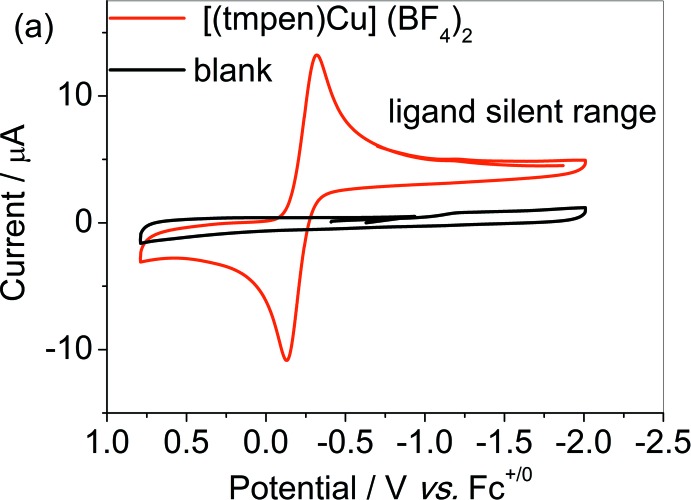
Cyclic voltammograms of complex **1** (1 m*M*) under Ar in CH_3_CN with 0.1 *M ^n^*Bu_4_NBF_4_ as the supporting electrolyte.

**Figure 3:**
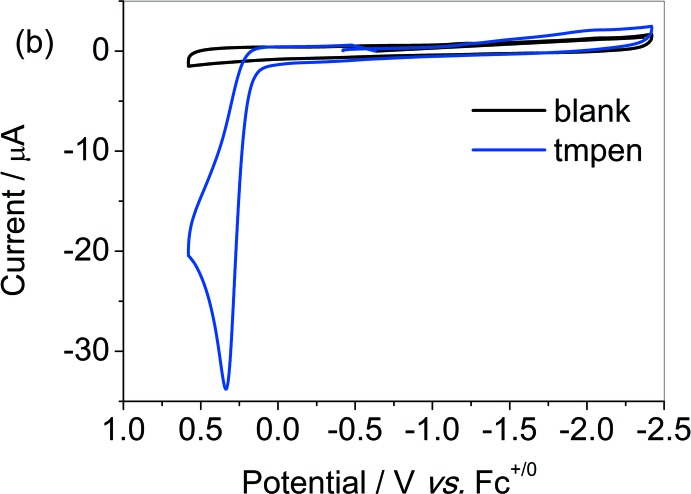
Cyclic voltammograms of the TMPEN ligand (1 m*M*) under Ar in CH_3_CN with 0.1 *M ^n^*Bu_4_NBF_4_ as the supporting electrolyte.

The distortion about the Cu^II^ atom is in favour of the reductive dissociation of one pyridine group. On a cathodic scan under Ar, complex **1** features one reversible couple based on copper at 0.26 V (*vs F*c^+/0^), assigned to Cu^II/I^ (Fig. 2 ▸). The free ligand tmpen is electrochemically silent in the potential range (Fig. 3 ▸). The good reversibility of the couple indicates negligible change in the configuation of **1** under reductive conditions.

## Supramolecular features   

**Figure 4:**
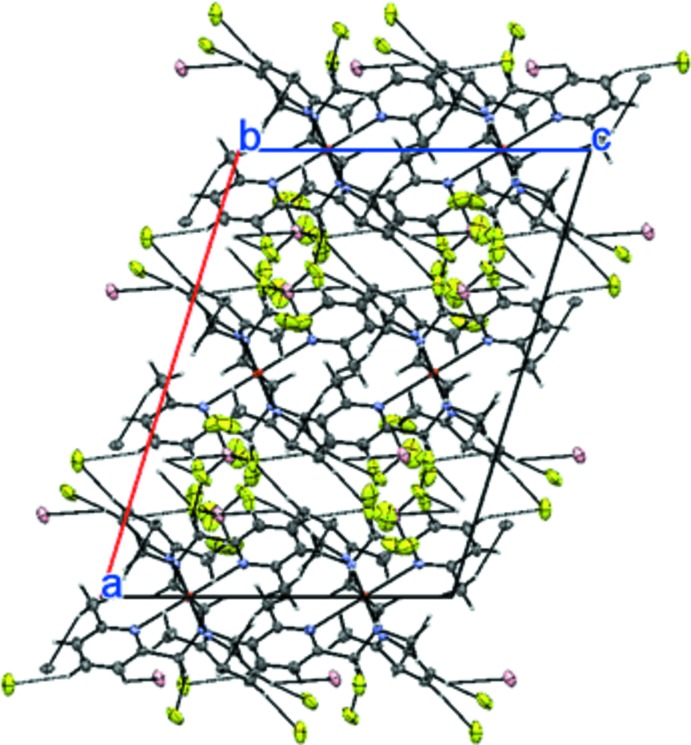
The crystal packing showing the C—H⋯F hydrogen bonds.

While there are no classical hydrogen bonds in the crystal structure, C—H⋯N and C—H⋯F inter­actions are observed (Fig. 4 ▸, Table 1 ▸).

## Database survey   

There are four published reports of polypyridine copper complexes (Kaur *et al.*, 2015 ▸; Meyer *et al.*, 2015 ▸; Bania & Deka, 2012 ▸; Yoon *et al.*, 2005 ▸) , but to the best of our knowledge, the title compound has not been reported previously. Among the earliest reports, the copper complex with an *N*,*N*,*N*′,*N*′-tetra­kis­(2-pyridyl­meth­yl)ethyl­enedi­amine (TPEN) ligand is most similar to title complex in configuration. In the presence of ascorbic acid as a reducing agent, Cu^2+^(TPEN) displays high activity in atom-transfer radical addition (ATRA) reactions (Kaur *et al.*, 2015 ▸). In contrast to Cu^2+^(TPEN), the title complex exhibits greater steric hindrance, which results in an evident Jahn–Teller effect on the configuration. In the title complex, the axial Cu—N bonds to pyridyl nitro­gen atoms [2.5742 (13) Å)] are significantly longer than in Cu^2+^(TPEN) [2.377 (3) and 2.308 (2) Å] while the differences in the equatorial Cu—N distances are negligible (Yoon *et al.*, 2005 ▸). The other two reported polypyridine copper complexes show similar distorted octa­hedral coordination spheres around the Cu^2+^ cation, but the ligands are evidently different from the title complex.

## Synthesis and crystallization   

The tetra­pyridinedi­amine ligand *N*,*N*,*N*′,*N*′-tetra­kis­[(6-methyl­pyridin-2-yl)meth­yl]ethane-1,2-di­amine (tmpen) was prepared according to literature procedures (Mikata *et al.*, 2005 ▸). ^1^H NMR (CDCl_3_, 600 MHz): δ 7.44 (*d*, 4H), 7.31 (*m*, 4H), 6.94 (*d*, 4H), 3.74 (*s*, 8H), 2.75 (*s*, 4H), 2.48 (*s*, 12H). ESI–MS: calculated for [*M* + H]^+^: *m*/*z* 481.65.19; found: 481.31.

For the preparation of [Cu(tmpen)](BF_4_)_2_ (**1**), Cu(BF_4_)_2_·H_2_O (0.16 g, 0.5 mmol) was added to an aceto­nitrile solution (5 ml) of tmpen (0.2 g, 0.5 mmol). The mixture was stirred at room temperature for 6 h. The blue solution was then transferred to tubes, which were placed in a flask containing ether. Block-shaped crystals were obtained in a yield of 85% (0.25 g). Analysis calculated for C_30_H_36_B_2_CuF_8_N_6_ (%): C, 50.52; H, 5.09; N, 11.78; found: 50.51; H, 5.08; N, 11.75; MS (TOF–ES): *m*/*z* =272.6641 {[*M* − 2(BF_4_)^−^]/2}^+^, 579.3025 [*M* − 2(BF_4_)^−^+Cl^−^]^+^.

## Refinement   

Crystal data, data collection and structure refinement details are summarized in Table 2 ▸. All F atoms of the BF_4_ group were split into two groups and their ccupancies determined *via* a free variable refinement. All hydrogen atoms were refined in riding mode with C—H= 0.93–0.97 and *U*_iso_(H) = 1.2*U*_eq_(C) or 1.5*U*_eq_(C) for methyl H atoms.

## Supplementary Material

Crystal structure: contains datablock(s) I. DOI: 10.1107/S2056989017004492/pj2042sup1.cif

Structure factors: contains datablock(s) I. DOI: 10.1107/S2056989017004492/pj2042Isup2.hkl

CCDC reference: 1440025

Additional supporting information:  crystallographic information; 3D view; checkCIF report

## Figures and Tables

**Table 1:** Hydrogen-bond geometry (Å, °)

*D*—H⋯*A*	*D*—H	H⋯*A*	*D*⋯*A*	*D*—H⋯*A*
C1—H1*B*⋯F2*A* ^i^	0.96	2.50	3.296 (17)	140
C4—H4*A*⋯F4*A* ^ii^	0.93	2.50	3.394 (15)	161
C5—H5*B*⋯F3^iii^	0.93	2.45	3.355 (9)	164
C5—H5*B*⋯F3*A* ^iii^	0.93	2.33	3.194 (13)	155
C7—H7*A*⋯N3^iv^	0.97	2.59	3.212 (2)	122
C8—H8*A*⋯F1*A* ^v^	0.97	2.48	3.298 (16)	142
C9—H9*A*⋯F3	0.97	2.55	3.436 (10)	152
C9—H9*B*⋯F4^v^	0.97	2.34	3.303 (6)	173
C12—H12*A*⋯F4^vi^	0.93	2.45	3.198 (7)	137

Symmetry codes: (i) 
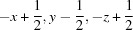
; (ii) 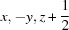; (iii) 
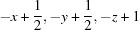
; (iv) 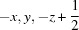; (v) 
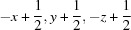
; (vi) 
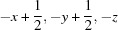
.

**Table 2:** Experimental details

Crystal data	
Chemical formula	[Cu(C_30_H_36_N_6_)](BF_4_)_2_
*M*_r_	717.81
Crystal system, space group	Monoclinic, *C*2/*c*
Temperature (K)	296
*a*, *b*, *c* (Å)	18.670 (2), 12.8309 (15), 14.0146 (16)
β (°)	107.193 (2)
*V* (Å^3^)	3207.2 (6)
*Z*	4
Radiation type	Mo *K*α
μ (mm^−1^)	0.76
Crystal size (mm)	0.30 × 0.20 × 0.10

Data collection	
Diffractometer	Bruker APEXII CCD area detector
Absorption correction	Multi-scan (*SADABS*; Bruker, 2013 ▸)
*T*_min_, *T*_max_	0.833, 0.927
No. of measured, independent and observed [*I* > 2σ(*I*)] reflections	10330, 3676, 3334
*R*_int_	0.023
(sin θ/λ)_max_ (Å^−1^)	0.649

Refinement	
*R*[*F* ^2^ > 2σ(*F* ^2^)], *wR*(*F* ^2^), *S*	0.034, 0.097, 1.06
No. of reflections	3676
No. of parameters	254
No. of restraints	40
H-atom treatment	H-atom parameters constrained
Δρ_max_, Δρ_min_ (e Å^−3^)	0.97, −0.25

Computer programs: *SMART* and *SAINT* (Bruker, 2013 ▸), *SHELXTL* (Sheldrick, 2008 ▸) and *SHELXL2014* (Sheldrick, 2015 ▸).

## supplementary crystallographic information

### Crystal data

**Table:**

[Cu(C_30_H_36_N_6_)](BF_4_)_2_	*F*(000) = 1476
*M**_r_* = 717.81	*D*_x_ = 1.487 Mg m^−^^3^*D*_m_ = 1.485 Mg m^−^^3^*D*_m_ measured by none
Monoclinic, *C*2/*c*	Mo *K*α radiation, λ = 0.71073 Å
*a* = 18.670 (2) Å	Cell parameters from 5092 reflections
*b* = 12.8309 (15) Å	θ = 2.3–27.5°
*c* = 14.0146 (16) Å	µ = 0.76 mm^−^^1^
β = 107.193 (2)°	*T* = 296 K
*V* = 3207.2 (6) Å^3^	Block, purple
*Z* = 4	0.30 × 0.20 × 0.10 mm

### Data collection

**Table:**

Bruker APEXII CCD area detector diffractometer	3334 reflections with *I* > 2σ(*I*)
Radiation source: sealed tube	*R*_int_ = 0.023
phi and ω scans	θ_max_ = 27.5°, θ_min_ = 2.0°
Absorption correction: multi-scan (SADABS; Bruker, 2013)	*h* = −12→24
*T*_min_ = 0.833, *T*_max_ = 0.927	*k* = −16→16
10330 measured reflections	*l* = −18→16
3676 independent reflections	

### Refinement

**Table:**

Refinement on *F*^2^	40 restraints
Least-squares matrix: full	Hydrogen site location: inferred from neighbouring sites
*R*[*F*^2^ > 2σ(*F*^2^)] = 0.034	H-atom parameters constrained
*wR*(*F*^2^) = 0.097	*w* = 1/[σ^2^(*F*_o_^2^) + (0.0549*P*)^2^ + 2.078*P*] where *P* = (*F*_o_^2^ + 2*F*_c_^2^)/3
*S* = 1.06	(Δ/σ)_max_ = 0.002
3676 reflections	Δρ_max_ = 0.97 e Å^−^^3^
254 parameters	Δρ_min_ = −0.25 e Å^−^^3^

### Special details

**Table:**

Geometry. All esds (except the esd in the dihedral angle between two l.s. planes) are estimated using the full covariance matrix. The cell esds are taken into account individually in the estimation of esds in distances, angles and torsion angles; correlations between esds in cell parameters are only used when they are defined by crystal symmetry. An approximate (isotropic) treatment of cell esds is used for estimating esds involving l.s. planes.

### Fractional atomic coordinates and isotropic or equivalent isotropic displacement parameters (Å^2^)

**Table:**

	*x*	*y*	*z*	*U*_iso_*/*U*_eq_	Occ. (<1)
Cu1	0.0000	0.23649 (2)	0.2500	0.02731 (10)	
N1	0.08743 (7)	0.15031 (11)	0.33863 (10)	0.0289 (3)	
N2	0.07204 (7)	0.35197 (10)	0.31608 (10)	0.0286 (3)	
N3	0.07474 (8)	0.26278 (11)	0.12413 (11)	0.0333 (3)	
C1	0.08575 (13)	0.00170 (16)	0.22917 (16)	0.0537 (6)	
H1A	0.0358	0.0242	0.1945	0.081*	
H1B	0.0863	−0.0726	0.2372	0.081*	
H1C	0.1190	0.0209	0.1914	0.081*	
C2	0.11081 (9)	0.05237 (13)	0.32937 (13)	0.0336 (4)	
C3	0.16060 (10)	0.00215 (15)	0.40983 (15)	0.0401 (4)	
H3A	0.1747	−0.0665	0.4036	0.048*	
C4	0.18899 (10)	0.05447 (16)	0.49875 (14)	0.0429 (4)	
H4A	0.2195	0.0201	0.5544	0.052*	
C5	0.17170 (10)	0.15836 (16)	0.50451 (13)	0.0387 (4)	
H5B	0.1938	0.1967	0.5620	0.046*	
C6	0.12094 (9)	0.20416 (14)	0.42312 (12)	0.0302 (3)	
C7	0.09995 (10)	0.31833 (13)	0.42177 (12)	0.0325 (3)	
H7A	0.0613	0.3280	0.4545	0.039*	
H7B	0.1434	0.3593	0.4569	0.039*	
C8	0.02775 (10)	0.45062 (13)	0.30188 (13)	0.0359 (4)	
H8A	0.0611	0.5101	0.3096	0.043*	
H8B	0.0012	0.4556	0.3517	0.043*	
C9	0.13719 (9)	0.36030 (14)	0.27541 (13)	0.0351 (4)	
H9A	0.1751	0.3108	0.3107	0.042*	
H9B	0.1585	0.4295	0.2903	0.042*	
C10	0.12107 (9)	0.34140 (14)	0.16498 (13)	0.0328 (4)	
C11	0.15943 (12)	0.39957 (17)	0.11282 (16)	0.0485 (5)	
H11A	0.1896	0.4552	0.1429	0.058*	
C12	0.15162 (15)	0.3728 (2)	0.01508 (17)	0.0619 (6)	
H12A	0.1770	0.4098	−0.0220	0.074*	
C13	0.10602 (14)	0.2910 (2)	−0.02692 (16)	0.0550 (6)	
H13A	0.1008	0.2714	−0.0925	0.066*	
C14	0.06769 (11)	0.23748 (15)	0.02882 (15)	0.0394 (4)	
C15	0.01701 (13)	0.14901 (19)	−0.01745 (17)	0.0551 (6)	
H15A	0.0011	0.1136	0.0331	0.083*	
H15B	0.0435	0.1012	−0.0475	0.083*	
H15C	−0.0260	0.1755	−0.0676	0.083*	
B1	0.31688 (17)	0.1726 (2)	0.2629 (2)	0.0555 (6)	
F1	0.3874 (4)	0.1486 (8)	0.3288 (7)	0.124 (2)	0.639 (19)
F2	0.3115 (7)	0.2722 (5)	0.2310 (10)	0.101 (3)	0.639 (19)
F3	0.2668 (5)	0.1626 (7)	0.3154 (7)	0.091 (2)	0.639 (19)
F4	0.3036 (5)	0.1037 (4)	0.1872 (4)	0.0811 (16)	0.639 (19)
F1A	0.3844 (7)	0.1315 (12)	0.2879 (15)	0.122 (3)	0.361 (19)
F4A	0.2738 (11)	0.1220 (13)	0.1767 (8)	0.119 (4)	0.361 (19)
F3A	0.2862 (10)	0.1559 (10)	0.3370 (10)	0.083 (3)	0.361 (19)
F2A	0.3273 (13)	0.2753 (11)	0.2471 (18)	0.107 (4)	0.361 (19)

### Atomic displacement parameters (Å^2^)

**Table:**

	*U*^11^	*U*^22^	*U*^33^	*U*^12^	*U*^13^	*U*^23^
Cu1	0.02415 (15)	0.02360 (15)	0.02934 (16)	0.000	0.00043 (11)	0.000
N1	0.0245 (6)	0.0307 (7)	0.0284 (6)	0.0006 (5)	0.0030 (5)	−0.0015 (5)
N2	0.0284 (6)	0.0289 (7)	0.0272 (6)	−0.0029 (5)	0.0063 (5)	−0.0041 (5)
N3	0.0315 (7)	0.0364 (8)	0.0341 (7)	−0.0066 (6)	0.0127 (6)	−0.0055 (6)
C1	0.0595 (13)	0.0401 (11)	0.0500 (12)	0.0127 (9)	−0.0017 (10)	−0.0144 (9)
C2	0.0275 (8)	0.0323 (8)	0.0385 (9)	0.0028 (6)	0.0058 (7)	−0.0014 (7)
C3	0.0311 (8)	0.0348 (9)	0.0503 (11)	0.0071 (7)	0.0061 (8)	0.0059 (8)
C4	0.0303 (9)	0.0528 (11)	0.0395 (9)	0.0074 (8)	0.0008 (7)	0.0110 (8)
C5	0.0310 (8)	0.0518 (11)	0.0294 (8)	0.0013 (8)	0.0031 (7)	−0.0006 (7)
C6	0.0251 (7)	0.0361 (8)	0.0280 (8)	−0.0007 (6)	0.0056 (6)	−0.0023 (6)
C7	0.0340 (8)	0.0359 (9)	0.0255 (8)	−0.0018 (7)	0.0054 (6)	−0.0055 (6)
C8	0.0412 (9)	0.0254 (8)	0.0396 (9)	−0.0019 (7)	0.0095 (8)	−0.0048 (7)
C9	0.0292 (8)	0.0410 (9)	0.0343 (8)	−0.0108 (7)	0.0083 (7)	−0.0063 (7)
C10	0.0295 (8)	0.0365 (9)	0.0332 (8)	−0.0044 (6)	0.0108 (7)	−0.0036 (7)
C11	0.0513 (11)	0.0490 (11)	0.0486 (11)	−0.0181 (9)	0.0199 (9)	−0.0020 (9)
C12	0.0711 (15)	0.0762 (16)	0.0472 (12)	−0.0258 (13)	0.0311 (11)	0.0008 (11)
C13	0.0630 (14)	0.0714 (15)	0.0368 (10)	−0.0142 (12)	0.0243 (10)	−0.0099 (10)
C14	0.0370 (9)	0.0468 (10)	0.0359 (9)	−0.0035 (7)	0.0134 (8)	−0.0100 (7)
C15	0.0571 (13)	0.0637 (14)	0.0487 (12)	−0.0175 (11)	0.0222 (10)	−0.0265 (10)
B1	0.0770 (18)	0.0415 (12)	0.0585 (15)	−0.0006 (12)	0.0363 (14)	−0.0085 (11)
F1	0.090 (3)	0.132 (5)	0.130 (5)	0.017 (3)	−0.001 (3)	−0.019 (3)
F2	0.130 (5)	0.043 (2)	0.123 (6)	−0.001 (3)	0.026 (4)	0.012 (3)
F3	0.086 (3)	0.109 (4)	0.105 (5)	−0.009 (2)	0.067 (3)	−0.023 (3)
F4	0.143 (4)	0.0526 (17)	0.0577 (18)	0.016 (2)	0.045 (2)	−0.0101 (15)
F1A	0.089 (5)	0.102 (5)	0.193 (10)	0.023 (4)	0.068 (6)	−0.033 (7)
F4A	0.169 (9)	0.102 (6)	0.085 (5)	−0.024 (6)	0.036 (5)	−0.057 (4)
F3A	0.146 (9)	0.059 (4)	0.061 (4)	−0.017 (5)	0.056 (5)	−0.011 (3)
F2A	0.166 (9)	0.057 (5)	0.097 (6)	−0.053 (5)	0.034 (7)	0.000 (4)

### Geometric parameters (Å, º)

**Table:**

Cu1—N2^i^	2.0311 (13)	C7—H7B	0.9700
Cu1—N2	2.0312 (13)	C8—C8^i^	1.516 (3)
Cu1—N1	2.0571 (13)	C8—H8A	0.9700
Cu1—N1^i^	2.0571 (13)	C8—H8B	0.9700
Cu1—N3	2.5742 (13)	C9—C10	1.506 (2)
Cu1—N3^i^	2.5742 (13)	C9—H9A	0.9700
N1—C2	1.349 (2)	C9—H9B	0.9700
N1—C6	1.354 (2)	C10—C11	1.384 (3)
N2—C7	1.482 (2)	C11—C12	1.378 (3)
N2—C9	1.492 (2)	C11—H11A	0.9300
N2—C8	1.493 (2)	C12—C13	1.370 (3)
N3—C10	1.342 (2)	C12—H12A	0.9300
N3—C14	1.343 (2)	C13—C14	1.387 (3)
C1—C2	1.492 (3)	C13—H13A	0.9300
C1—H1A	0.9600	C14—C15	1.497 (3)
C1—H1B	0.9600	C15—H15A	0.9600
C1—H1C	0.9600	C15—H15B	0.9600
C2—C3	1.390 (2)	C15—H15C	0.9600
C3—C4	1.376 (3)	B1—F1A	1.315 (11)
C3—H3A	0.9300	B1—F3A	1.343 (10)
C4—C5	1.379 (3)	B1—F4	1.347 (6)
C4—H4A	0.9300	B1—F2	1.347 (7)
C5—C6	1.380 (2)	B1—F3	1.356 (6)
C5—H5B	0.9300	B1—F2A	1.360 (12)
C6—C7	1.515 (2)	B1—F4A	1.398 (10)
C7—H7A	0.9700	B1—F1	1.401 (6)
			
N2^i^—Cu1—N2	86.31 (8)	H7A—C7—H7B	108.4
N2^i^—Cu1—N1	165.41 (5)	N2—C8—C8^i^	108.82 (11)
N2—Cu1—N1	79.43 (6)	N2—C8—H8A	109.9
N2^i^—Cu1—N1^i^	79.43 (6)	C8^i^—C8—H8A	109.9
N2—Cu1—N1^i^	165.41 (5)	N2—C8—H8B	109.9
N1—Cu1—N1^i^	114.97 (8)	C8^i^—C8—H8B	109.9
N1—Cu1—N3	89.43 (5)	H8A—C8—H8B	108.3
N1—Cu1—N3^i^	98.67 (5)	N2—C9—C10	116.30 (13)
N2—Cu1—N3	78.28 (5)	N2—C9—H9A	108.2
N2—Cu1—N3^i^	90.69 (5)	C10—C9—H9A	108.2
N3—Cu1—N3^i^	164.94 (5)	N2—C9—H9B	108.2
C2—N1—C6	118.72 (14)	C10—C9—H9B	108.2
C2—N1—Cu1	131.62 (11)	H9A—C9—H9B	107.4
C6—N1—Cu1	109.44 (11)	N3—C10—C11	123.31 (16)
C7—N2—C9	108.49 (13)	N3—C10—C9	117.86 (15)
C7—N2—C8	113.42 (13)	C11—C10—C9	118.59 (16)
C9—N2—C8	111.74 (13)	C12—C11—C10	118.13 (19)
C7—N2—Cu1	103.48 (10)	C12—C11—H11A	120.9
C9—N2—Cu1	112.51 (10)	C10—C11—H11A	120.9
C8—N2—Cu1	106.97 (10)	C13—C12—C11	119.23 (19)
C10—N3—C14	117.87 (15)	C13—C12—H12A	120.4
C2—C1—H1A	109.5	C11—C12—H12A	120.4
C2—C1—H1B	109.5	C12—C13—C14	119.71 (19)
H1A—C1—H1B	109.5	C12—C13—H13A	120.1
C2—C1—H1C	109.5	C14—C13—H13A	120.1
H1A—C1—H1C	109.5	N3—C14—C13	121.70 (18)
H1B—C1—H1C	109.5	N3—C14—C15	118.54 (17)
N1—C2—C3	120.78 (16)	C13—C14—C15	119.76 (18)
N1—C2—C1	118.43 (15)	C14—C15—H15A	109.5
C3—C2—C1	120.68 (17)	C14—C15—H15B	109.5
C4—C3—C2	119.65 (17)	H15A—C15—H15B	109.5
C4—C3—H3A	120.2	C14—C15—H15C	109.5
C2—C3—H3A	120.2	H15A—C15—H15C	109.5
C3—C4—C5	119.35 (17)	H15B—C15—H15C	109.5
C3—C4—H4A	120.3	F1A—B1—F3A	108.9 (10)
C5—C4—H4A	120.3	F4—B1—F2	112.5 (7)
C4—C5—C6	118.58 (17)	F4—B1—F3	111.6 (5)
C4—C5—H5B	120.7	F2—B1—F3	105.9 (6)
C6—C5—H5B	120.7	F1A—B1—F2A	105.1 (10)
N1—C6—C5	122.16 (16)	F3A—B1—F2A	113.3 (10)
N1—C6—C7	115.57 (13)	F1A—B1—F4A	107.8 (6)
C5—C6—C7	122.26 (15)	F3A—B1—F4A	109.0 (8)
N2—C7—C6	107.90 (12)	F2A—B1—F4A	112.5 (12)
N2—C7—H7A	110.1	F4—B1—F1	107.0 (4)
C6—C7—H7A	110.1	F2—B1—F1	113.0 (7)
N2—C7—H7B	110.1	F3—B1—F1	106.6 (5)
C6—C7—H7B	110.1		
			
C6—N1—C2—C3	9.0 (2)	C7—N2—C8—C8^i^	−152.37 (17)
Cu1—N1—C2—C3	−165.01 (13)	C9—N2—C8—C8^i^	84.61 (19)
C6—N1—C2—C1	−167.28 (18)	Cu1—N2—C8—C8^i^	−38.93 (19)
Cu1—N1—C2—C1	18.7 (3)	C7—N2—C9—C10	151.06 (15)
N1—C2—C3—C4	−2.9 (3)	C8—N2—C9—C10	−83.17 (18)
C1—C2—C3—C4	173.28 (19)	Cu1—N2—C9—C10	37.18 (18)
C2—C3—C4—C5	−4.6 (3)	C14—N3—C10—C11	2.4 (3)
C3—C4—C5—C6	5.7 (3)	C14—N3—C10—C9	−171.80 (17)
C2—N1—C6—C5	−7.8 (2)	N2—C9—C10—N3	−41.5 (2)
Cu1—N1—C6—C5	167.40 (14)	N2—C9—C10—C11	143.95 (18)
C2—N1—C6—C7	171.25 (15)	N3—C10—C11—C12	−2.4 (3)
Cu1—N1—C6—C7	−13.52 (17)	C9—C10—C11—C12	171.8 (2)
C4—C5—C6—N1	0.5 (3)	C10—C11—C12—C13	0.6 (4)
C4—C5—C6—C7	−178.54 (16)	C11—C12—C13—C14	0.9 (4)
C9—N2—C7—C6	−73.22 (16)	C10—N3—C14—C13	−0.7 (3)
C8—N2—C7—C6	162.00 (13)	C10—N3—C14—C15	179.19 (19)
Cu1—N2—C7—C6	46.47 (14)	C12—C13—C14—N3	−0.9 (4)
N1—C6—C7—N2	−22.45 (19)	C12—C13—C14—C15	179.2 (2)
C5—C6—C7—N2	156.64 (16)		

Symmetry code: (i) −*x*, *y*, −*z*+1/2.

### Hydrogen-bond geometry (Å, º)

**Table:**

*D*—H···*A*	*D*—H	H···*A*	*D*···*A*	*D*—H···*A*
C1—H1*B*···F2*A*^ii^	0.96	2.50	3.296 (17)	140
C4—H4*A*···F4*A*^iii^	0.93	2.50	3.394 (15)	161
C5—H5*B*···F3^iv^	0.93	2.45	3.355 (9)	164
C5—H5*B*···F3*A*^iv^	0.93	2.33	3.194 (13)	155
C7—H7*A*···N3^i^	0.97	2.59	3.212 (2)	122
C8—H8*A*···F1*A*^v^	0.97	2.48	3.298 (16)	142
C9—H9*A*···F3	0.97	2.55	3.436 (10)	152
C9—H9*B*···F4^v^	0.97	2.34	3.303 (6)	173
C12—H12*A*···F4^vi^	0.93	2.45	3.198 (7)	137

Symmetry codes: (i) −*x*, *y*, −*z*+1/2; (ii) −*x*+1/2, *y*−1/2, −*z*+1/2; (iii) *x*, −*y*, *z*+1/2; (iv) −*x*+1/2, −*y*+1/2, −*z*+1; (v) −*x*+1/2, *y*+1/2, −*z*+1/2; (vi) −*x*+1/2, −*y*+1/2, −*z*.
